# Labor Market Affiliation of Marginal Part-Time Workers in Denmark—A Longitudinal Study

**DOI:** 10.3390/ijerph19137634

**Published:** 2022-06-22

**Authors:** Helena Breth Nielsen, Kathrine Pape, Laura Stonor Gregersen, Jonas Kirchheiner-Rasmussen, Johnny Dyreborg, Anna Ilsøe, Trine Pernille Larsen, Jacob Pedersen, Anne Helene Garde

**Affiliations:** 1 The National Research Centre for the Working Environment, 2100 Copenhagen, Denmark; kpape@live.dk (K.P.); laura_gregersen@hotmail.com (L.S.G.); xjor@nfa.dk (J.K.-R.); jdy@nfa.dk (J.D.); jpe@nfa.dk (J.P.); ahg@nfa.dk (A.H.G.); 2 Employment Relations Research Centre (FAOS), University of Copenhagen, 1353 Copenhagen, Denmark; ai@faos.dk (A.I.); tpl@faos.dk (T.P.L.); 3 Department of Public Health, University of Copenhagen, 1353 Copenhagen, Denmark

**Keywords:** working hours, full-time workers, unemployment, long-term sickness absence, social benefits, students

## Abstract

This longitudinal study examined the labor market affiliations of marginal part-time workers (<15 working hours/week) compared with full-time workers (32–40 working hours/week) within gender and age groups. Analyses were based on 1,492,187 Danish employees with marginal part-time or full-time work at baseline using register data of working hours and labor market affiliation from the Labor Market Account. We used the Expected Labor Market Affiliation method within gender and age groups to estimate the time spent in different labor market states over a 5-year follow-up from 2012–2017. The multistate model included five recurrent labor market states: work, unemployment, long-term sickness absence, studying, and temporarily out, and the results were adjusted for education level, morbidity, and ethnicity. A marginal part-time worker generally had fewer days of work without social benefits and spent more days studying during follow-up compared with a full-time worker. In addition, marginal part-time workers ≥ 25 years old had more days of unemployment and more days of long-term sickness absence. These findings suggest that marginal part-time workers have fewer paid workdays without social benefits compared with full-time workers, depending on age. Further studies should explore whether marginal part-time work is a stepping stone into or out of the labor market.

## 1. Introduction

The Danish labor market has been called a “flexicurity model”, as it combines flexibility in hiring and layoffs for employers and social protection for employees [1]. Wages and working conditions are largely regulated through collective agreements within each sector between employers’ organizations and unions, and there is high collective agreement coverage [2]. The majority of Danish employees work full-time, with a norm of 37 h of work per week. In 2020, around 40% of the Danish workforce reported having 37 h of work per week based on questionnaire data [3]. Another 30% of the Danish workforce reported more than 37 h, and 30% reported less than 37 h of work per week [3]. Marginal part-time work is a form of atypical employment, which has increased in the Danish labor market since 2000 [4]. In 2020, around 10 % of the Danish workforce were marginal part-time workers [3]. Although no universal definition of marginal part-time work exists, it is often described as working less than 15 h per week [5,6]. Marginal part-time workers in Denmark are typically young people, women, low-skilled workers, and students and are widespread within the sectors: hotels and restaurants, arts and entertainment, and retail [7,8,9]. In a previous study on marginal part-time workers, we found that marginal part-time workers more often had services and sales jobs, while full-time workers were more often professionals [10].

There can be different reasons for taking on marginal part-time work. Marginal part-time work may be a way to earn some extra money while studying or be a way into the labor market for newcomers, individuals with patchy careers, or people with reduced workability due to chronic disease. Marginal part-time work can also act as a stepping stone into the labor market after unemployment [11]. In Denmark, the majority of marginal part-time workers report school or training as their main reason for having marginal part-time work [9]. Other reasons include illness or disability or difficulties in finding a full-time job [9]. These reasons are likely gender- and age-related and could also influence the subsequent labor market affiliation.

Marginal part-time workers experience lower job security, fewer career opportunities, less training investment by employers, and lower pay compared to their open-ended full-time contract counterparts [9,12,13]. A recent Danish study showed that marginal part-time workers reported poorer self-rated health than full-time workers [10]. They also generally report a poorer work environment compared with full-time workers [10,14,15]. Several of these aspects have been linked to the risk of mental disorders and long-term sickness absence [16,17,18,19], which may in the long run lead to poorer labor market affiliation. However, only a few studies have addressed the labor market affiliations of marginal part-time workers and have so far only been limited to the case of disability pension [20,21]. A Norwegian register-based cohort study of 14,657 participants in 2002 found that working <20 h/week increased the risk of disability pension [20]. This was confirmed in a Swedish cohort study of 17,640 part-timers in 2014 working <50 % full-time [21].

In this study, we aimed to follow labor market affiliations over a five-year period, from 2012–2017, for marginal part-time workers (<15 h/week) compared with full-time workers (32–40 h/week) at baseline in 2012 within gender and age groups. To the best of our knowledge, no studies have yet investigated marginal part-time workers’ future labor market affiliations by assessing possible labor market transitions or the number of days in different labor market states (e.g., work, long-term sickness absence, and unemployment). Understanding marginal part-time workers’ labor market affiliations within gender and age groups will provide insights into whether or how these groups of marginal part-time workers are differentiated from full-time workers in the labor market. This may help identify target groups for preventive strategies, e.g., to increase re-employment rates or reduce the number of days of long-term sickness absence.

## 2. Materials and Methods

Nationwide register-based data from Statistics Denmark were linked by the unique personal identification numbers assigned to all residents of Denmark [22].

### 2.1. Study Design

This longitudinal study used unique data on working hours from the Labor Market Account without standardization of hours (AMR-UN) [23]. The AMR-UN contains information on labor market affiliations from 2008 and onwards of all Danish citizens with less than 4% imputed paid hours of work for employees in 2013 [24]. The quality of the AMR-UN is considered to be high [24]. Daily information on working hours, based on tax payments, and payment of all major social benefits, e.g., unemployment and long-term sickness absence benefits, are registered on an individual level in the AMR-UN, which is composed of several national administrative registers [25]. We further linked this information to registers at Statistics Denmark, including the Population Education Register [26], the Income Statistics Register [27], the Employment Classification Module [28], the Danish National Patient Register [29], and the Danish Register of Cause of Death [30].

### 2.2. Study Population

**Figure 1:**
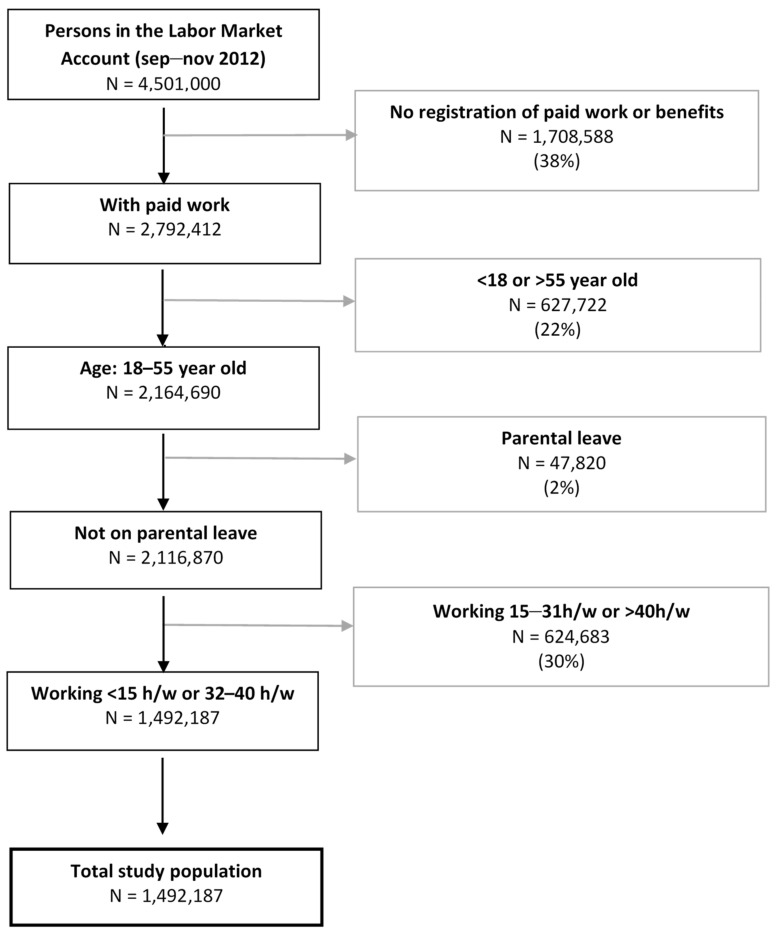
Flowchart of the study population. h/w = hours per week.

We included all Danish employees (with at least 1 h of paid work) between 18 and 55 years old at baseline in 2012. The age cut-off was chosen to only include a working population during the 5-year follow-up, as the early retirement age in Denmark was 61.5 years of age until 2016 [31]. We included those who had less than 15 h of paid work/week or between 32–40 h of work/week on average for the previous three months before baseline but excluded those on parental leave. The total study population consisted of 1,492,187 employees in Denmark. The selection of the study population is illustrated in Figure 1.

### 2.3. Marginal Part-Time Work (Exposure)

Marginal part-time work was assessed by using data from the AMR-UN with information on working hours by employed persons (employees, self-employed, or assisting spouses). The average number of weekly working hours across a period of three months, from 1 September to 30 November 2012, was calculated for each employee. Workers could receive other benefits at the same time, e.g., unemployment or student benefits, during this period. Marginal part-time work was defined as <15 h of work per week, based on previously used definitions of marginal part-time [9,32]. The reference group with full-time work was defined as 32.0–40.0 h per week on average for the same period of three months, around the full-time norm in Denmark, which is 37 h/week.

### 2.4. Labor Market Affiliation (Outcome)

Labor market affiliation was assessed by using eight labor-market states, longitudinally obtained from the AMR-UN, hereunder defined as five recurrent states: (1) work—salary payments, without any social benefits; (2) unemployment—persons available for the labor market receiving social benefits for unemployment; (3) long-term sickness absence—persons receiving sickness absence benefits (entitled after >30 consecutive days of sickness); (4) students—persons who receive benefits for undergoing education; and (5) temporarily out—persons on parental leave and periods without any registered salary or benefits; and three absorbing states: (1) disability retirement pension, (2) voluntary retirement pension, and (3) death or emigration, in line with a previous study [33] on labor market affiliation. See Appendix B Table A1 for detailed codes. Absorbing states, where transitions are not possible after entry, leading to being out of the labor market, were controlled for (see Appendix C Table A2 for the full model). For the recurrent states, multiple and recurrent transitions were possible. The states in the model were mutually exclusive. In the case of multiple registrations on the same day, the states were prioritized as follows: absorbing states (death or emigration, disability retirement pension state, or voluntary retirement pension state) over recurrent states, which were prioritized as: (1) long-term sickness absence, (2) student, (3) unemployment, (4) temporarily out, and lastly, (5) work. Thus, in the analyses, individuals receiving student benefits were classified as a student regardless of the number of paid work hours. Consequently, the work state exclusively included days without any social benefits. Labor market affiliation was followed for five years (corresponding to a maximum of 1826 days), from 1 December 2012 to 1 December 2017, to limit major fluctuations in the labor market over longer periods.

**Figure 2:**
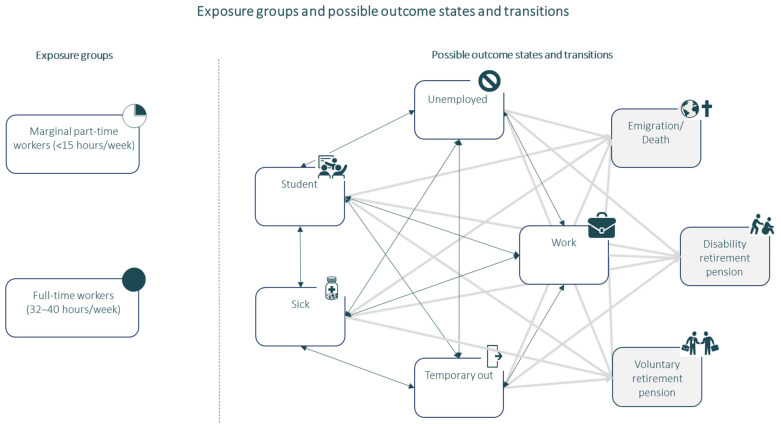
Exposure groups and possible outcome states and transitions. Work = Salary payments, without any social benefits, Temporary out = Parental leave and periods without any registered income, Unemployment = Person available for the labor market receiving social benefit for unemployment, Student = Persons under education, Sickness absence = Long-term sick-listed (>30 consecutive days) receiving sickness absence benefits, Voluntary retirement pension = Time after voluntary retirement (censoring state), Disability retirement pension (Including flex job) = Time after awarded disability pension due to e.g. chronic illness (absorbing state), Emigration or death (censoring state). 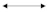 = Two-way transition. 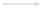 = One-way transition. 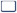 = recurrent stage. 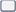 = absorbing/censoring state.

A multistate model with boxes illustrating the possible recurrent and absorbing states is shown in Figure 2.

### 2.5. Covariates

**Figure A1:**
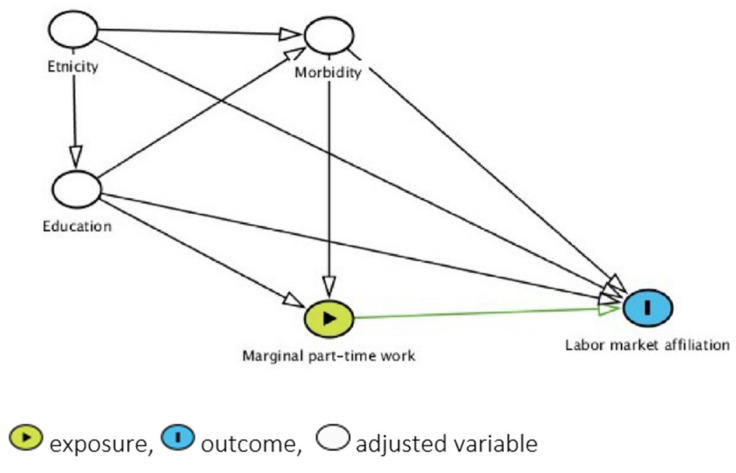
Directed acyclic graph of marginal part-time work and labor market affiliation in gender and age groups.

Analyses were adjusted for potential confounders identified a priori from the current literature [9,21,34] and based on a directed acyclic graph (see Appendix A Figure A1). The identified potential confounders were highest attained education [35] (primary/secondary/higher), morbidity [21,34,36] (none/≥1 diagnosis), and ethnicity [9,37,38] (Danish origin/immigrant/descendant). Morbidity was assessed by the Charlson Comorbidity Index based on information from the Danish National Patient Register [29,39,40] during the past five years. Information on the potential confounders was obtained on 30 November 2012 (baseline year) from national registers at Statistics Denmark.

### 2.6. Statistics

Transition probabilities for labor market affiliations for marginal part-time and full-time workers were analyzed with multistate modeling according to the Expected Labor Market Affiliation (ELMA) method [33] developed by Pedersen (2021). Participants were followed from 1 December 2012 to the end of the study period (1 December 2017), pension, emigration, or death, whichever came first (right censoring). To estimate the average number of days in the different labor market states, we first estimated the transition and state probabilities for each transition and state in the multistate model. This was carried out separately on marginal part-time and full-time workers, using days from the start of follow-up as the underlying time axis. Then, we summed the area under each transition and state probability, as conducted in Pedersen et al. 2021. Finally, to test the difference between marginal part-time and full-time workers, a variance regression model was conducted on 500 re-samples, assuming a normally distributed state duration. The analysis was performed separately within gender (women/men) and age groups: 18–24, 25–34, 35–44, and 45–55 years old. We used inverse probability weights with the highest attained education, morbidity, and ethnicity to adjust for covariates. All analyses were conducted using SAS V.9.4 software (SAS Institute Inc., Cary, NC, USA).

## 3. Results

The study population had a mean age of 38 (standard deviation 10.7) years, and 47% were women. In Table 1, the characteristics of marginal part-time workers and full-time workers at baseline are presented. Compared with full-time workers, marginal part-time workers were younger, more often women, less educated, slightly more often previously diagnosed, and more often immigrants or descendants.

**Figure 3:**
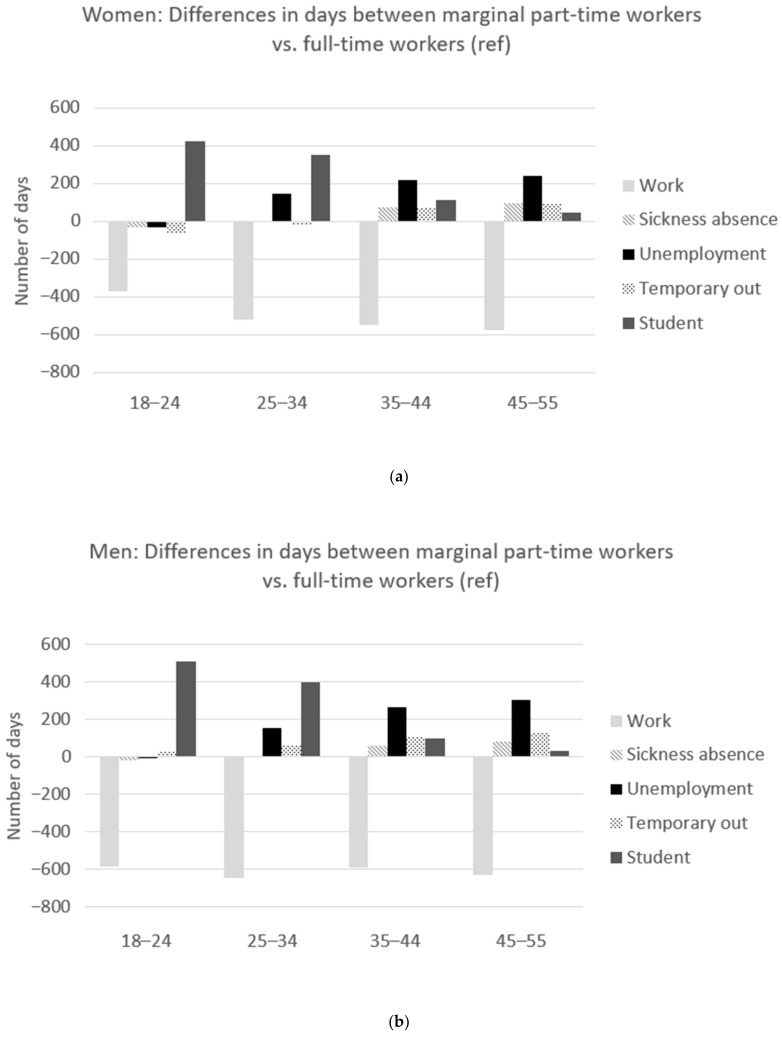
The difference between the estimated days in the recurrent states for marginal part-time workers compared with full-time workers (reference) over the five years of follow-up by age group and in women (**a**) and men (**b**). Days represent periods within a given state, which also includes days off (e.g., weekends). Figure 3 shows that a marginal part-time worker at baseline in the following five years is estimated to have between 370–647 fewer days of work without any social benefits compared with a full-time worker at baseline. Instead, a marginal part-time worker is estimated to spend more days as a student. In addition, marginal part-time workers ≥ 25 years old are also estimated to have more days of unemployment, long-term sickness absence, and being temporarily out of the labor market.

In Table 2, the estimated days in the labor market states during a five-year follow-up are shown for marginal part-time workers and full-time workers stratified by gender and age groups. Figure 3 illustrates the five labor market states and the estimated difference in days between a marginal part-time worker and a full-time worker in women (Figure 3a) and men (Figure 3b) and by age group. In the following, the results are described separately for each labor market state but are interrelated, as the states were mutually exclusive. Thus, more days in one state would mean fewer days in the other states during the five-year follow-up.

### 3.1. Days of Work

Compared with a full-time worker at baseline, a marginal part-time worker at baseline generally had fewer days of work over the five years of follow-up than a full-time worker (see Table 2). Over the five-year follow-up, a woman with marginal part-time work was estimated to have between 370 and 576 fewer days of work across the age groups compared with a woman who was working full-time at baseline (see Figure 3a). For men, the numbers were between 585 and 647 fewer days of work across the age groups (see Figure 3b).

### 3.2. Days of Long-Term Sickness Absence

Among both men and women, a marginal part-time worker was estimated to have more days of long-term sickness absence during follow-up compared with a full-time worker (see Table 2). The results indicated that the additional number of days of long-term sickness absence among marginal part-time workers increased with age (see Figure 3). Thus, a marginal part-time working woman had up to 99 more estimated sick days and a man had up to 82 more sick days than a full-time worker. However, in the youngest age group, marginal part time-workers were estimated to have fewer long-term sickness absence days than full-time workers (women −28 days, and men −19 days).

### 3.3. Days Unemployed

Marginal part-time workers were estimated to spend more days unemployed in the five-year follow-up period compared with full-time workers, except in the youngest age group (see Table 2). The results indicated higher numbers of additional days of unemployment among marginal part-time workers with increasing age (see Figure 3). Marginal part-time workers above 24 years old spent between 147 and 243 more days unemployed among women and 155–304 more days unemployed among men compared with the corresponding groups of full-time workers. In the youngest age groups (18–24 years old), women with marginal part-time work had fewer estimated unemployment days (−28 days), while no difference was detected among men.

### 3.4. Days Temporarily out of the Labor Market

Marginal part-time working men had between 28 and 127 more days temporarily out of the labor market than full-time working men across the age groups (see Figure 3b). Women older than 34 years old with marginal part-time work spent between 74 and 92 estimated more days temporarily out of the labor market than full-time time working women, whereas women younger than 35 years old spent between 61 and 17 fewer estimated days temporarily out of the labor market (see Figure 3a).

### 3.5. Days as a Student

Across all age groups, a marginal part-time worker was estimated to spend more days as a student than full-time workers (see Table 2). Marginal part-time working women were estimated to spend between 47 and 427 more days as a student, and marginal part-time working men had between 31 and 513 more days compared with full-time workers during follow-up (see Figure 3). The largest differences were seen in the youngest age group, and the difference fell with increasing age for both men and women.

## 4. Discussion

In this study, we followed marginal part-time workers’ labor market affiliations over a five-year period within gender and age groups. We found that a marginal part-time worker, compared with a full-time worker at baseline, in the following five years had fewer days of work without any social benefits and more days as a student. Except for the youngest age group, a marginal part-time worker also experienced more days of unemployment, long-term sickness absence, and being temporarily out of the labor market.

To the best of our knowledge, no previous studies have addressed the labor market affiliations of marginal part-time workers by assessing the time spent in different labor market states compared with full-time workers. However, in the Nordic countries and Germany, marginal part-time work has been related to other negative labor market aspects, such as higher job insecurity, higher income insecurity, and a discrepancy between desired and experienced security and development in the job [9,10,41]. The overall finding of fewer days of work among marginal part-time workers is interconnected with the findings of marginal part-time workers spending more days studying and, dependent on age, days of long-term sickness absence, unemployment, and being temporarily out in the five-year follow-up period.

### 4.1. Marginal Part-Time Workers below 25 Years of Age

Half of all marginal part-time workers (52%) were found in the youngest age group, from 18 to 24 years old. In this group, marginal part-time workers generally had more days as students, fewer days of work, and fewer days of long-term sickness absence during follow-up compared with full-time workers. This suggests that many of the marginal part-time workers in the youngest age group have marginal part-time work in student jobs, which is supported by the fact that young marginal part-time workers in Denmark are often students [9]. In Denmark, education is free, and Danish students are entitled to a monthly grant from the Danish government to help cover living costs [42]. This reduces the financial need for a full-time job, and among students in higher education with a job on the side, more than 90% work less than 15 h per week [43]. Our finding of more days spent as a student among marginal part-time workers compared with full-time workers is supported by a Danish study, which found that the main reason for having marginal part-time work is undergoing school education or training [9]. In our model, the time during which a person receives student grants is categorized as being a student, despite possible (marginal part-time) work during follow-up. Thus, the de facto number of work days in the student category is assumed to be higher. In terms of long-term sickness absence, a higher education level is associated with less sickness absence [35]. Therefore, the fewer days of sickness absence among marginal part-time workers in the youngest age group may also be related to a larger share of students among marginal part-time workers compared with full-time workers.

In the young age group, marginal part-time work may serve as a stepping stone into the labor market. Certain aspects of marginal part-time work can be argued to be precarious (for instance, contracts with no guaranteed hours), and a Danish study found that for some young workers, having precarious work is related to a transition from education to work life, yet for others, there is a risk of ending up in precarious work permanently [44]. Thus, young workers may also experience marginal part-time work differently according to their job and position in the labor market [44].

### 4.2. Marginal Part-Time Workers from 25 Years of Age and Above

In the age groups between 25 and 55 years old, our results indicated a general trend of more sick days with increasing age, possibly related to the general decline in self-rated health with age in working-aged adults [45]. Our results of more days of long-term sickness absence among marginal part-time workers ≥ 25 years old are supported by studies suggesting poorer health among marginal part-time workers compared with full-time workers [10,14,15]. In addition, marginal part-time work is associated with higher job insecurity [10], and job insecurity has been related to higher risks of mental disorders and long-term sickness absence [16,17,18,19]. Furthermore, high job insecurity, connected to a higher risk of job loss [46], is also consistent with marginal part-time workers having more or longer periods of unemployment, as also observed in the present study. Some marginal part-time workers in Denmark may struggle to qualify for unemployment benefits and statutory sick pay, even if they are a member of an unemployment benefit fund, due to a minimum threshold of 1924 accrued working hours within the last 3 years to qualify for unemployment benefits if a full-time insured member (1258 accrued working hours if part-time insured). Thus, marginal part-time workers risk receiving less generous entitlements [4]. As such, marginal part-time work may include aspects that are unfavorable for the employee.

In the older age groups, less time was spent studying during follow-up, and with increasing age, the difference in days as a student between marginal part-time workers and full-time workers decreased. Instead, our results indicated that with increasing age, marginal part-time workers had more additional days of long-term sickness absence and unemployment compared with the group of full-time workers. The tendency of more additional days of long-term sickness absence with older age in marginal part-time workers may be explained by workers with health problems choosing marginal part-time work instead of staying in, or going into, full-time jobs. Thus, in some instances, marginal part-time work could be a stepping stone into the labor market or a way to stay in the labor market for those who cannot hold a full-time job for health reasons.

### 4.3. Men and Women in Marginal Part-Time Work

Our results generally showed similar patterns for marginal part-time working women and men. However, the results indicated larger differences in days of work between marginal part-time workers and full-time workers among men than women. This may be explained by slightly more additional days as a student, days of unemployment, and in particular, days of being temporarily out among marginal part-time working men.

Altogether, the population of marginal part-time workers appears to be a heterogeneous group of workers across age groups that shift from largely consisting of students in the young age group to a more vulnerable group of workers with more health problems in the older age groups. Previous results on marginal part-time workers also indicated that students with marginal part-time work reported better health and less job insecurity compared with other marginal part-time workers in Denmark [10]. In the young age group with a larger share of students, marginal part-time work may act as a stepping stone into the labor market, while in the older age group, marginal part-time work may be a way to hold on to a job. These hypotheses need to be explored in future studies.

### 4.4. Strengths and Limitations

A major strength of the study is the large nationwide sample size with objective and detailed longitudinal register-based data covering different sectors and job titles in several labor markets states. The use of nationwide register-based data limits both the risk of selection bias and attribution bias, as all workers were followed up. Moreover, the use of register measures of both the exposure and outcome eliminates the risk of recall bias. Nevertheless, some misclassification may be present due to unregistered hours or fluctuations in weekly work hours. Work days are based on tax payments and are therefore sensitive to undeclared work. Undeclared work is illegal in Denmark, yet around 20% of the 18–74-year-olds in the Danish population were estimated to be engaged in undeclared work in 2017 [47]. However, the median amount of undeclared work was one hour per week [47], and tax is primarily subtracted by the employer before payment; thus, we expect little misclassification in the exposure measure. In addition, there is a financial incentive to register the other labor market states, e.g., long-term sickness absence and studying, as the workplace or the individual may be entitled to a social benefit. It is only possible to be in a single state at a time in the multistate model. In the case of registrations of two states at the same time, e.g., some workers were both studying and working at the same time, the states were prioritized. This prioritization was chosen to be able to show how many marginal part-time workers received some sort of social benefit compared to full-time workers. The prioritizing described in the Methods section will therefore have an impact on the interpretation of the results. It is especially important to note that the work state includes only time with paid work and without any social benefits, which is why the number of actual workdays is underestimated. This may be particularly true for those who were marginal part-time workers because these were also more often students and therefore also represented in more states. Concerning the absorbing states, there is a risk of overestimating the time spent in, for example, the disability pension state, as we did not include the risk of further events, e.g., death. Therefore, the time in the absorbing states should be considered with some caution. Additionally, we did not examine the possibility of pensioning other than the voluntary pension scheme. In addition, multiple job-holding or multiple income holding was not captured separately. Selection effects are likely to be present; e.g., in the young group of marginal part-time workers, a larger share were students at baseline and continued to be students during the follow-up. Thus, the design limits inferences on causal effects of marginal part-time work but does not rule them out. Instead, these results represent the time that a marginal part-time worker is expected to spend in different labor market states during a five-year follow-up.

The ELMA method provides a comprehensive and easy overview of the complex pattern of transitions in the multistate model, as shown in Figure 2. In several studies, a similar methodology has proven to provide solid estimates on duration when taking a life course perspective [48,49,50]. However, like other predictive modeling studies, the results should be adjusted if the underlying circumstances change, e.g., economic situations impacting the dynamics of the labor market. The possibility of stratifying and adjusting for numerous factors is a strength.

One limitation of the study is the risk of reverse causation, particularly regarding sick days [51], although the longitudinal design reduced this risk. Hence, the finding of more sick days among marginal part-time workers could be a result of underlying illness rather than marginal part-time work in itself. The adjustment of results for morbidity using weights, in addition to education and ethnicity, reduced this possible confounding factor. Another limitation when it comes to sick days is that marginal part-time workers do not always qualify for full-wage compensation whilst on sick leave. Paid sick leave is guaranteed in most collective agreements, but marginal part-time workers often work in areas that are not covered by collective agreements and tend not to be members of an unemployment benefit fund, and even if this is the case, they often struggle to meet the working hours threshold for acquiring statutory sick pay [4]. This would lead to fewer registered sick days among marginal part-time workers. Thus, the actual number of additional sick days among marginal part-time workers may be even higher in the older age groups.

Our findings may be generalized with caution to other Nordic countries with similar flexicurity welfare state models and social contexts, as different rules and regulations in a particular country may impact the labor market states [14,52,53]. It is also important to consider the time period when generalizing these findings, as other factors, e.g., economic fluctuations, may affect the results. We propose that further studies use a longitudinal design when studying labor market affiliations of marginal part-time workers and look closer at identifying different groups with marginal part-time work to evaluate whether marginal part-time work serves as a stepping stone or a dead-end job. In addition, further studies may consider separating short- and long-term effects.

## 5. Conclusions

This study provides new insights into marginal part-time workers’ labor market affiliations over a five-year follow-up period and highlights variations by age groups, which appears to be an important factor to consider in future studies on marginal part-time workers. Marginal part-time workers appear to be a heterogeneous group of workers. A young marginal part-time worker spent more additional days as a student in the following five years compared with a full-time worker, while an older marginal part-time worker spent more additional days temporarily out of the labor market and had more days of long-term sickness absence and unemployment. These insights into how the group of marginal part-time workers differentiates compared with full-time workers and within age strata can help to identify target groups for preventive strategies, e.g., to facilitate re-employment. Further follow-up studies are needed to understand whether marginal part-time work is a stepping stone into or out of the labor market, with specifications by different age groups. Further research could aim at separating short- and long-term effects and identifying different groups with marginal part-time work.

## Figures and Tables

**Table 1:** Characteristics of (*n* = 1,492,187) employees in Denmark working marginal part-time (<15 h per week) or full-time (32–40 h per week) between 1 September and 30 November 2012.

	Marginal Part-Time		Full-Time		Total		*p*-Value
	*n*	%	*n*	%	*n*	%	
Total	290,299	19	1,201,888	81	1,492,187	100	
**Age**							<0.0001
18–24	152,215	52	90,934	8	243,149	16	
25–34	65,645	23	265,232	22	330,877	22	
35–44	34,353	12	396,856	33	431,209	29	
45–55	38,086	13	448,866	37	486,952	33	
Missing	0	0	0	0	0	0	
**Gender**							<0.0001
Women	162,322	56	540,241	45	702,563	47	
Men	127,977	44	661,647	55	789,624	53	
Missing	0	0	0	0	0	0	
**Highest attained** **education**							<0.0001
Primary	103,308	36	185,313	15	288,621	19	
Secondary	133,803	46	608,223	51	742,026	50	
Higher	35,363	12	381,492	32	416,855	28	
Missing	17,825	6	26,860	2	44,685	3	
**Morbidity**							<0.0001
Registered diagnosis past 5 years	10,579	4	41,094	3	51,673	3	
**Ethnicity**							<0.0001
Danish origin	245,149	84	1,094,013	91	1,339,162	90	
Immigrant	34,249	12	93,990	8	128,239	9	
Descendant	9021	3	10,139	1	19,160	1	
Missing	1880	1	3746	0	5626	0	

*n* = number of employees. % = percentage of employees. *p*-values are based on Chi2 tests. Note: Table 1 presents characteristics of marginal part-time workers and full-time workers at baseline in 2012. Compared with full-time workers, marginal part-time workers were more often: between 18–24 years old, women, less educated, previously diagnosed, and immigrants or descendants.

**Table 2:** Numbers of days in different labor market states during the five years of follow-up by gender and age groups.

**Women**																				
**Age**	**18–24**					**25–34**					**35–44**					**45–55**				
	**Marginal Part-Time**		**Full-Time**		***p*** **-Value**	**Marginal Part-Time**		**Full-Time**		***p*** **-Value**	**Marginal Part-Time**		**Full-Time**		***p*** **-Value**	**Marginal Part-Time**		**Full-Time**		***p*** **-Value**
**States**	**Days**	**95% CI**	**Days**	**95% CI**		**Days**	**95% CI**	**Days**	**95% CI**		**Days**	**95% CI**	**Days**	**95% CI**		**Days**	**95% CI**	**Days**	**95% CI**	
Work	444	438–450	814	803–826	<0.001	776	763–788	1295	1289–1301	<0.001	1034	1016–1053	1580	1577–1583	<0.001	1059	1040–1079	1635	1633–1638	<0.001
Sickness absence	15	13–16	43	40–47	<0.001	59	54–64	52	50–54	0.004	133	124–143	59	57–60	<0.001	162	151–174	63	62–65	<0.001
Unemployment	83	80–86	111	105–117	<0.001	206	198–215	59	57–61	<0.001	264	251–277	42	40–43	<0.001	286	272–300	43	42–44	<0.001
Temporarily out	94	91–97	155	148–162	<0.001	205	197–214	222	218–226	<0.001	118	109–128	44	42–45	<0.001	106	97–116	14	13–14	<0.001
Student	1060	1053–1066	633	622–644	<0.001	519	508–529	165	162–169	<0.001	198	187–210	84	82–86	<0.001	98	89–107	51	50–53	<0.001
**Men**																				
**Age**	**18–24**					**25–34**					**35–44**					**45–55**				
	**Marginal Part-Time**		**Full-Time**		***p*** **-Value**	**Marginal Part-Time**		**Full-Time**		***p*** **-Value**	**Marginal Part-Time**		**Full-Time**		***p*** **-Value**	**Marginal Part-Time**		**Full-Time**		***p*** **-Value**
**States**	**Days**	**95% CI**	**Days**	**95% CI**		**Days**	**95% CI**	**Days**	**95% CI**		**Days**	**95% CI**	**Days**	**95% CI**		**Days**	**95% CI**	**Days**	**95% CI**	
Work	499	492–507	1084	1075–1093	<0.001	876	862–889	1523	1519–1527	<0.001	1073	1051–1094	1664	1661–1666	<0.001	1042	1021–1063	1672	1670–1674	<0.001
Sickness absence	10	9–12	29	27–32	<0.001	35	31–39	28	27–30	0.003	88	79–99	30	29–32	<0.001	123	112–134	41	40–43	<0.001
Unemployment	81	77–85	82	78–86	0.856	204	195–213	49	47–51	<0.001	303	288–320	37	35–38	<0.001	349	332–366	45	44–46	<0.001
Temporarily out	81	78–85	53	50–56	<0.001	106	100–114	46	45–48	<0.001	137	125–149	26	25–28	<0.001	147	136–160	20	19–21	<0.001
Student	1030	1023–1038	517	509–526	<0.001	542	531–554	144	141–146	<0.001	147	136–160	50	48–51	<0.001	58	48–63	27	26–28	<0.001

Days represent the estimated number of days within a given state, which also includes days off (e.g., weekends). Results are weighted for education, ethnicity, and comorbidity. 95% CI = 95 % confidence intervals. *p*-value = Chi2 tests based on a variance regression model with five hundred re-samples, assuming a normally distributed state duration. The full table with absorbing states is shown in Appendix C Table A2. Note: Table 2 presents the estimated number of days in the recurrent labor market states over a five-year period among marginal part-time and full-time workers and by gender and age groups. Results show that a marginal part-time worker at baseline is estimated to have fewer days of work without any social benefits and more days as a student in the following five years compared with a baseline full-time worker. In addition, except in the youngest age group, a marginal part-time worker is estimated to have more days of unemployment, long-term sickness absence, and being temporarily out of the labor market.

## Data Availability

The anonymized micro data used for this study are available from Statistics Denmark. Permission of access to the data can only be granted through an affiliation with a Danish authorized environment. For more information on data and data access, please visit www.dst.dk/en/TilSalg/Forskningsservice (accessed on 1 January 2022).

## Appendix B

**Table A1:** Coding of labor market affiliations (outcome variables).

**Work and social benefits:** Codes in the Labor Market Account (from the variable: SOC_STATUS_KODE_T_A) to define the labor market states (disability pension, early retirement pension, work, unemployment, sickness benefits, temporarily out, and student benefits).		
**Absorbing states:** Codes in the Danish Civil Registration System (emigration) and the Cause of Death Register (death).		
**Variables**	**Codes**	**Description**
Censoring states		
**Death**		Cause of Death Register (D_DODSDTO before 2014 and D_DODSDATO after 2014)
**Emigration**		The Danish Civil Registration System (indud_land, indud_kode and haend_dato)
Absorbing states		
**Disability pension**	314	Unemployment benefit (Ledighedsydelse)
	351	Flexible job subsidy (Fleksløntilskud)
	411	Disability pension (Førtidspension)
	413	Flexible job benefits (Fleksydelse)
**Voluntary** **retirement pension**	412	Early retirement benefit (Efterløn)
**Censoring**	414	Retirement pension (Folkepension)
	415	Other pension (Anden pension)
Recurrent states		
**Work**	110	Self-employed, main state at the end of November (Selvstændige, primær status ult. nov.)
	111	Self-employed, secondary state at the end of November (Selvstændige, sekundær status ult. nov.)
	120	Co-working spouse, main state (Medarbejdende ægtefæller, primær status ult. nov.)
	121	Co-working spouse, secondary state (Medarbejdende ægtefæller, sekundær status ult. nov)
	131	Salaried employee in managerial work (Topledere)
	132	Salaried employee in employment that imply high-level skills (Lønmodtagere på højeste niveau)
	133	Salaried employee in employment that imply mid-level skills (Lønmodtagere på mellemniveau)
	134	Salaried employee in employment that imply ground level skills (Lønmodtagere på grundniveau)
	135	Other salaried employed (Andre lønmodtagere)
	136	Salaried employed, unspecified (Lønmodtagere u.n.a.)
	137	Salaried employed at the end of November not main employment (Lønmodtager ult. nov. ikke primære job)
	138	Salaried employed not at the end of November (Lønmodtager ej ult. Nov)
	312	Holiday allowance (Feriedagpenge)
**Unemployment**	200	Unemployed (Arbejdsløse)
	311	Supported employments without pay (Støttet beskæftigede uden løn)
	313	Guidance and upgrading education (Vejledning og opkvalificering)
	318	Social assistance (Kontanthjælp, passiv)
	319	Introductory benefit for foreigners (Introduktionsydelse)
	320	Rehabilitation benefit (Revalidering)
	321	Rehabilitation programme (Ressourceforløb)
**Sickness benefit**	612	Sickness absence employment, not main state (Sygefravær fra beskæftigelse, ej primær status)
	317	Sick absence unemployment
	322	Job clarification course (Jobafklaringsforløb)
**Temporarily out**	315	Leave of absence for childcare, unemployed (Børnepasningsorlov fra ledighed)
	316	Maternity benefit, unemployed (Barselsfravær fra ledighed)
	512	Children and adolescents (Børn og unge)
	513	Others out of the labor force (Øvrige uden for arbejdsstyrken)
	611	Maternity leave from employment, not main state (Barselsfravær fra beskæftigelse, ej primær status)
**Student benefit**	511	Ongoing education, regularly (Personer under uddannelse, ordinær)
	514	Course participant (Kursister)
	515	Student at a production school (Produktionsskoleelever)
	516	Foreign students (Udenlandske studerende, ud fra opholdsgrundlag)
	517	State education grant (Modtagere af SU)

## Appendix C

Full Table 2, including absorbing states.

**Table A2:** Numbers of days in different labor market states during the five years of follow-up by gender and age groups.

**Women**																				
**Age**	**18–24**					**25–34**					**35–44**					**45–55**				
	**Marginal Part-Time**		**Full-Time**		***p*** **-Value**	**Marginal Part-Time**		**Full-Time**		***p*-Value**	**Marginal Part-Time**		**Full-Time**		***p*-Value**	**Marginal Part-Time**		**Full-Time**		***p*-Value**
**States**	**Days**	**95% CI**	**Days**	**95% CI**		**Days**	**95% CI**	**Days**	**95% CI**		**Days**	**95% CI**	**Days**	**95% CI**		**Days**	**95% CI**	**Days**	**95% CI**	
Disability retirement	1	0–2	1	1–2	<0.001	7	6–10	2	2–3	<0.001	37	32–43	4	4–5	<0.001	67	60–74	8	7–8	<0.001
Voluntary retirement	0	0–0	0	0–0	1.000	0	0–0	0	0–0	1.000	0	0–0	0	0–0	1.000	0	0–0	0	0–0	1.000
Death or emigration	51	49–53	24	22–1827	<0.001	38	35–42	19	18–20	<0.001	11	8–14	6	6–10	0.006	15	12–19	5	5–5	<0.001
Men																				
**Age**	**18–24**					**25–34**					**35–44**					**45–55**				
	**Marginal Part-Time**		**Full-Time**		***p*-Value**	**Marginal Part-Time**		**Full-Time**		***p*-Value**	**Marginal Part-Time**		**Full-Time**		***p*-Value**	**Marginal Part-Time**		**Full-Time**		***p*-Value**
**States**	**Days**	**95% CI**	**Days**	**95% CI**		**Days**	**95% CI**	**Days**	**95% CI**		**Days**	**95% CI**	**Days**	**95% CI**		**Days**	**95% CI**	**Days**	**95% CI**	
Disability retirement	1	1–2	1	1–2	0.193	6	4–9	1	1–2	<0.001	27	22–33	3	2–3	<0.001	58	51–66	6	6–7	<0.001
Voluntary retirement	0	0–0	0	0–0	1.000	0	0–0	0	0–0	1.000	0	0–0	0	0–0	1.000	0	0–0	0	0–0	1.000
Death or emigration	45	43–48	24	22–359	<0.001	44	40–1827	24	23–26	<0.001	24	19–29	11	10–589	<0.001	19	15–24	9	8–9	<0.001

Weighted for education, ethnicity, and comorbidity. Days represent periods within a given state, which also includes days off (e.g., weekends). *p*-value = Chi2 tests based on a variance regression model with five hundred re-samples, assuming a normally distributed state duration.
